# The prognostic significance of inflammation-based scores in patients with ampullary carcinoma after pancreaticoduodenectomy

**DOI:** 10.1186/s12885-020-07482-0

**Published:** 2020-10-10

**Authors:** Shuxin Sun, Chaobin He, Jun Wang, Xin Huang, Jiali Wu, Shengping Li

**Affiliations:** Department of Pancreatobiliary Surgery, State Key Laboratory of Oncology in South China, Collaborative Innovation Center for Cancer Medicine, Sun Yat-sen University Cancer Center, 651 dongfengdong Road, Guangzhou, 510060 China

**Keywords:** Neutrophil-to-lymphocyte ratio, Prognostic index, Disease-free survival, Overall survival, Ampullary cancer, Pancreaticoduodenectomy

## Abstract

**Background:**

Growing evidence indicates that the systemic inflammatory response plays an important role in cancer development and progression. Several inflammatory markers have been reported to be associated with clinical outcomes in patients with various types of cancer. This study was designed to evaluate the prognostic value of inflammatory indexes in patients with ampullary cancer (AC) who underwent pancreaticoduodenectomy (PD).

**Methods:**

We retrospectively reviewed the data of 358 patients with AC who underwent PD between 2009 and 2018. R software was used to compare the area under the time-dependent receiver operating characteristic (ROC) curves (AUROCs) of the inflammation-based indexes, including the neutrophil-to-lymphocyte ratio (NLR), platelet-to-lymphocyte ratio (PLR), modified Glasgow Prognostic Score (mGPS), prognostic nutritional index (PNI) and prognostic index (PI), in terms of their predictive value for survival. The survival differences of these indexes were compared by the Kaplan-Meier method and univariate and multivariate analyses were performed to determine the prognostic factors of disease-free survival (DFS) and overall survival (OS).

**Results:**

The estimated 1-, 2-, and 3-year OS and DFS rates were 83.9, 65.8, and 55.2% and 58.0, 42.8, and 37.8%, respectively, for the entire cohort. The survival differences were significant in terms of OS and DFS when patients were stratified by these inflammation-based indexes. The comparisons of the AUROCs of these inflammation-based indexes illustrated that NLR and PI displayed the highest prognostic value, compared to the other indexes. When NLR and PI were combined, NLR-PI showed even higher AUROC values and was identified as a significant prognostic factor for OS and DFS.

**Conclusion:**

Specific inflammatory indexes, such as NLR, PLR and dNLR, were found to be able to predict the OS or DFS of patients. As a novel inflammatory index, the level of NLR-PI, which can be regarded as a more useful prognostic index, exhibited strong predictive power for predicting the prognosis of patients with AC after the PD procedure.

## Background

Malignancies arising within 2 cm of the major papilla in the duodenum are defined as periampullary cancers (PACs), which generally encompass four different anatomical subtypes: pancreatic, ampullary, biliary, and duodenal cancers [1]. Ampullary cancer (AC) is the second most common type of PAC, accounting for approximately 16 to 28% of PACs [2–4]. Due to the specific anatomical structure and biliary obstruction, distinctive clinical symptoms usually arise in patients at an early stage, so operative treatments are available. Typically, pancreaticoduodenectomy (PD) is selected for treatment [5]. However, partly because either chemotherapy or radiotherapy only has limited efficacy, the majority of patients eventually experience recurrent disease [6].

Due to the lack of large-scale prospective studies, it is difficult to accurately predict the prognosis of AC patients treated with the Whipple procedure. Although several studies have reported that few parameters, such as the symptoms and general state of the patients, tumour size, pathological grade, lymphatic metastasis and carbohydrate antigen 19–9 (CA19–9) levels, are related to the survival rate, they were not sufficiently powerful. Therefore, a better predictive index for the survival time and risk of recurrence of patients with postoperative ampullary carcinoma is essential.

It is now well accepted that the immune and inflammatory response of cancer patients has a close relationship with the development and progression of malignancies [7, 8]. After Virchow first described the presence of leukocytes in tumour tissue in 1863, the connection between inflammation and cancer has drawn great attention in various malignancies [9]. The current understanding suggests that inflammation-based indexes, such as the neutrophil-to-lymphocyte ratio (NLR) and platelet-to-lymphocyte ratio (PLR), could be promising indicators for the prognosis [10, 11]. Lv et al. reported that the preoperative NLR level was able to predict prognosis in patients with glioblastoma, and Kim et al. confirmed that NLR, together with the Glasgow prognostic score and serum level of PIVKA, offered significant prognostic information associated with early recurrence for hepatocellular carcinoma patients with liver cirrhosis after curative resection [12, 13].

Nevertheless, for patients with AC who underwent PD as curative resection, the number of studies on the predictive ability of inflammation-based indexes is still small. In this study, we aimed to assess the prognostic value of a comprehensive systemic inflammatory factors, including NLR, PLR, prognostic index (PI), modified Glasgow prognostic score (mGPS) and prognostic nutritional index (PNI), and compare the predictive power of these indexes for predicting the overall survival (OS) or disease-free survival (DFS) of the AC patients treated with the Whipple procedure. More importantly, when we innovatively combined NLR with PI as a novel inflammation-based score, NLR-PI showed a superior discriminative capacity.

## Methods

### Patients

A series of 358 patients were enrolled in this study. Patients who underwent PD as curative resection and had histopathologically confirmed AC after resection at the Sun Yat-sen University Cancer Center between January 2009 and December 2018 were enrolled.

More specifically, the inclusion criteria were as follows: (1) all patients with a histopathological diagnosis of adenocarcinoma anatomically located in the ampulla of Vater; (2) patients who received blood routine analyses before and after surgery; and (3) patients who underwent PD according to standard surgical procedures. The exclusion criteria were as follows: (1) patients who were diagnosed with carcinoid histopathologically; (2) patients with a diagnosis of second tumours; and (3) patients who were lost to follow-up. No neoadjuvant chemotherapy was administered in these patients. There were 192 (53.6%) patients who had not received adjuvant chemotherapy, and another 166 (46.4%) patients who had received adjuvant chemotherapy. A uniformed chemotherapy regimen (gemcitabine- based chemotherapy) was used for these patients.

### Clinical management

Conventional therapeutic treatment was performed for each patient. Based on the medical examination results, including computed tomography (CT) or magnetic resonance (MR) imaging, blood biochemistry, tumour biomarker levels, and endoscopic ultrasonography-guided fine-needle aspiration (EUS-FNA), once the patients were diagnosed with AC and the tumour was resectable for the Whipple procedure, standard PD was performed, and treatment with suitable adjuvant therapy followed. Among the entire cohort, the average time from the date of blood collection to surgery was approximately 2 days.

### Clinical data extraction

Serological examination and radiological and clinicopathologic factors that were potentially associated with survival and recurrence were selected in this study, including age, sex, tumour markers CA19–9 and carcinoembryonic antigen (CEA), tumour diameter and location, pathological pattern, white blood cell count, platelet (PLT) count, neutrophil cell count, lymphocyte cell count, aspartate transaminase (AST), alanine transaminase (ALT), total bilirubin, gamma-glutamyl transpeptidase (GGT), albumin (ALB), C-reactive protein (CRP), NLR, derived neutrophil-to-lymphocyte ratio (dNLR), PLR, PI and so on. The 8th edition of the American Joint Committee on Cancer (AJCC) TNM staging system was adopted. Clinical and radiological data at the time of diagnosis and before and after the operation were retrieved [8, 14]. All of the inflammation-based prognostic scores determined in this study are described in Table 1.

**Table 1:** Inflammation-based prognostic scores

Scoring systems	Score
NLR	
Neutrophil count:lymphocyte count ≤3.32	0
Neutrophil count:lymphocyte count > 3.32	1
PLR	
Platelet count:lymphocyte count ≤99.02	0
Platelet count:lymphocyte count > 99.02	1
dNLR	
Neutrophil count:lymphocyte count ≤1.94	0
Neutrophil count:lymphocyte count > 1.94	1
PI	
CRP (≤10 mg/L) and WBC (≤11 × 10^9^/L)	0
CRP (≤10 mg/L) and WBC (>11 × 10^9^/L)	1
CRP (>10 mg/L) and WBC (≤11 × 10^9^/L)	1
CRP (>10 mg/L) and WBC (>11 × 10^9^/L)	2
mGPS	
CRP (≤10 mg/L) and ALB (≥35 g/L)	0
CRP (≤10 mg/L) and ALB (<35 g/L)	0
CRP (>10 mg/L) and ALB (≥35 g/L)	1
CRP (>10 mg/L) and ALB (<35 g/L)	2
PNI	
ALB (g/L) × total lymphocyte count × 10^9^/L ≥ 45	0
ALB (g/L) × total lymphocyte count × 10^9^/L<45	1

*NLR* Neutrophil-to-lymphocyte ratio, *PLR* Platelet-to-lymphocyte ratio, *dNLR* Derived neutrophil-to-lymphocyte ratio, *PI* Prognostic index, *mGPS* Modified glasgow prognostic score, *WBC* White blood cell counts, *CRP* C-reactive protein, *PNI* Prognostic nutritional index, *ALB* Albumin

### Follow-up

After discharge from the hospital, all patients were followed up at least once every 3 months during the first year and once every 6 months thereafter. Routine blood examinations serological examinations, and imaging examinations were selectively performed as needed. A routine follow-up was conducted by the follow-up department of Sun Yat-Sen University Cancer Center. OS was defined as the time from surgery to death from any cause or censorship at the date of the last follow-up. DFS was calculated from the time of surgery to the date of tumour progression discovered for the first time or death. There were 15 patients who were lost to follow-up in this study. The follow-up rate was more than 95%.

### Optimal cutoff values for the variables

The NLR score was calculated by dividing the neutrophil counts by the lymphocyte counts. The PLR score was calculated by dividing the platelet counts by the lymphocyte counts. The dNLR score was calculated by dividing the neutrophil counts by the white blood cell counts minus the neutrophil counts. The optimal cut-off values for the NLR, PLR and dNLR scores were determined using time-dependent ROC analysis. The NLR, PLR and dNLR scores were associated with the highest Youden index for the OS and DFS prediction, with cut-off values of 3.32, 1.94 and 99.02 respectively. The threshold for each clinical and radiological dataset was utilized as the cut-off value for these variables.

### Statistical analysis

Statistical analysis was performed using SPSS version 22 (SPSS Inc., Chicago, IL, USA). Continuous data are expressed as the means and ranges, and categorical data are shown as frequencies and proportions. Student’s t-test was used to compare continuous variables. The chi-squared test and Fisher’s exact test were used to compare the categorical variables. Univariate analysis was performed to assess the significance of clinical and radiological characteristics. Multivariate analysis was performed using the Cox regression model for variables found to be significant in univariate analysis, and the corresponding 95% confidence intervals (CIs) were calculated. The Kaplan–Meier method was used to analyse OS. Significant differences between the groups were identified using the log-rank test. The survival curves were generated using MedCalc software version 11.4.2.0 (http://www.medcalc.be). A two-tailed *P*-value < 0.05 was considered statistically significant.

Time-dependent receiver operating characteristic (ROC) curves were calculated to determine the optimal cutoff values and to assess the predictive power of these inflammation-based indexes in predicting long-term survival [15, 16]. The analyses of ROC curves and comparisons of the areas under the ROC curves (AUROCs) were performed using R software version 3.2.2 (The R Foundation for Statistical Computing, Vienna, Austria. http://www.r-project.org) with the “survival ROC” package and the “survival ROC.C” package.

## Results

### Patient characteristics

The baseline characteristics of the patients are summarized in Table 2. A total of 358 patients who were diagnosed with AC and underwent the Whipple procedure as curative resection were included in the final analysis. The median age was 58 years (range: 25 to 85) and there were 216 (60.3%) male patients and 142 (39.7%) female patients in the whole study cohort. Additionally, according to the TNM staging criteria, 165 patients (46.1%) were in stage I, 138 patients (38.6%) were in stage II, and 55 patients (15.4%) were in stage III. Furthermore, 159 (44.4%) patients were sorted into the lymphatic metastasis group. The median values of lymphocytes, neutrophils, and platelets were 1.60 ×  10^9^/L (range: 0.4 × 10^9^/L to 4.7 × 10^9^/L), 4.4 × 10^9^/L (range: 1.3 × 10^9^/L to 9.6 × 10^9^/L) and 305.56 × 10^9^/L (range: 84 × 10^9^/L to 720 × 10^9^/L), respectively. Moreover, the tumour differentiation degree was classified into well (7, 2.0%), moderate (188, 52.5%) and poor (163, 45.5%) differentiation,166 (46.4%) patients underwent chemotherapy.

**Table 2:** Clinical and radiological characteristics of the study cohort

Characteristics	Parameter	N (%)
**Age (years)**	<60/ ≥60	212 (59.2)/146 (40.8)
**Gender**	Female / Male	142 (39.7) /216 (60.3)
**WBC(× 10**^**9**^**/L)**	<10/≥10	329 (91.9) /29 (8.1)
**Neutrophil count(×10**^**9**^**/L)**	<7/≥7	289 (80.7) /69 (19.3)
**Lymphoeyte count(×10**^**9**^**/L)**	<4/≥4	348 (97.2)/10 (2.8)
**HGB(g/L)**	<100/≥100	170 (47.5) /188 (52.5)
**PLT(×10**^**9**^**/L)**	<300/≥300	191 (53.4) /167 (46.6)
**ALT(U/L)**	<40/≥40	91 (25.4)/267 (74.6)
**AST(U/L)**	<45/≥45	96 (26.8) /262 (73.2)
**ALB(g/L)**	<35/≥35	70 (19.6) /288 (80.4)
**TBIL (mmol/L)**	<20.5/≥20.5	80 (22.3) /278 (77.7)
**IBIL (mmol/L)**	<15/≥15	182 (50.8) /176 (49.2)
**CRP (mg/L)**	<8/≥8	108 (30.2) /250 (69.8)
**mGPS**	0 / 1 / 2	222 (62.0) /87 (24.3) /49 (13.7)
**CA19–9(U/ml)**	<35/≥35	90 (25.1) /268 (74.9)
**CEA (μg/L)**	<5/≥5	261 (72.9) / 97 (27.1)
**TNM**	IA/IB/IIA/IIB/III	72 (20.1) / 93 (26.0) / 34 (9.5) / 104 (29.1) / 55 (15.4)
**LN metastasis**	absent/present	199 (55.6) /159 (44.4)
**Tumor differentiation**	high/ moderate / poor	7 (2.0) / 188 (52.5) / 163 (45.5)
**NLR**	≤3.32 />3.32	226 (63.1) /132 (36.9)
**PLR**	≤99.02/>99.02	31 (8.7) / 327 (91.3)
**dNLR**	≤1.94/>1.94	204 (57.0)/154 (43.0)
**mGPS**	0/1/2	222 (62.0) /87 (24.3) /49 (13.7)
**PNI**	0 / 1	277 (77.4) /81 (22.6)
**PI**	0 / 1 / 2	216 (60.3) / 119 (33.2) / 23 (6.4)
**Chemotherapy**	No / Yes	192 (53.6) / 166 (46.4)

*HGB* Hemoglobin, *PLT* Platelets, *ALT* Alanine aminotransferase, *AST* Aspartate aminotransferase, *TBIL* Total serum bilirubin, *IBIL* Indirect serum bilirubin, *AFP* Alpha-fetoprotein, *CA19–9* Carbohydrate antigen 19–9, *TNM* Tumour-node-metastasis, *CEA* Carcinoembryonic antigen, *LN* Lymph node. Other Abbreviations as in Table 1

The NLR, PLR and dNLR scores were divided into two groups: ≤ 3.32 and > 3.32, ≤ 99.02 and > 99.02, and ≤ 1.94 and > 1.94, respectively. Among the 358 patients, 132 (36.9%) patients had an elevated NLR score; 327 (91.3%) patients had an elevated PLR score; 154 (43.0%) patients had an elevated dNLR score; 136 (38%) patients had an mGPS > 0; 277 (77.4%) patients had PNI ≥45; and 142 (39.6%) patients were allocated to PI 1 or 2.

### OS and prognostic factors

**Fig. 1:**
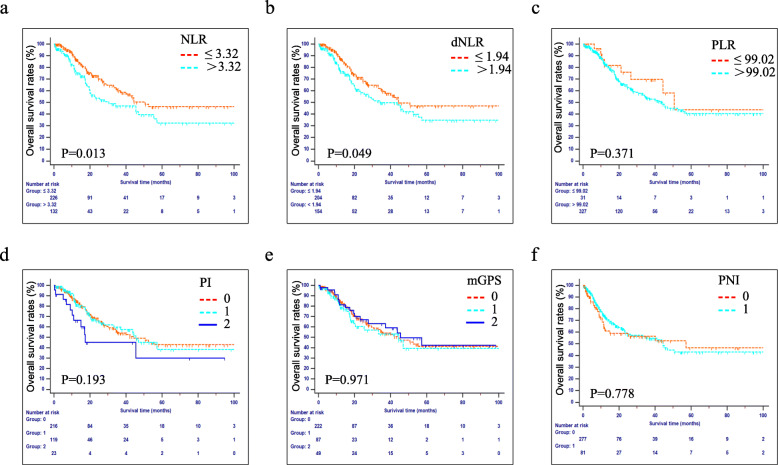
Kaplan-Meier curves of the OS in patients who were diagnosed with AC after PD. Patients were stratifed according to each inflammation-based index. **a** NLR, **b** dNLR, **c** PLR, **d** PI, **e** mGPS and **f** PNI

The median OS for the entire cohort was 44.3 months and the estimated 1-, 2-, and 3-year OS rates were 83.9, 65.8, and 55.2%, respectively. The long-term survival rates were significantly higher for patients with lower NLR values than for those with higher NLR values (*P* < 0.05, Fig. 1a). Moreover, patients with dNLR ≤1.94 also had better long-term survival than patients with dNLR > 1.94 (*P* < 0.05, Fig. 1b). However, other inflammatory indexes, including PLR, PI, mGPS and PNI, cannot be used to distinguish the long-term survival rates of patients in either of the respective groups (Fig. 1c-f).

In univariate survival analysis, NLR and dNLR were significantly associated with OS (NLR: hazard ratio (HR) 1.599, 95% CI 1.104–2.317, *P* < 0.05; dNLR: HR 1.451, 95% CI 1.002–2.101, *P* < 0.05). Other significant prognostic parameters included age, neutrophilic granulocyte count, IBIL, tumour differentiation, macrovascular or microvascular invasion, lymph node metastasis, TNM stage, CEA, CA19–9 and lymph node metastasis stage. According to the multivariate Cox proportional hazards model, tumour differentiation can be viewed as an independent prognostic indicator of OS (HR 1.669, 95% CI 1.126–2.476, *P* < 0.05) (Table 3).

**Table 3:** Univariate and multivariate analyses of OS

Characteristic	Parameter	Univariate analysis		Multivariate analysis	
		HR(95% CI)	***P***	HR(95% CI)	***P***
**Gender**	Female / Male	0.712 (0.492–1.032)	0.073	–	NI
**Age** (years)	<60/ ≥60	1.570 (1.083–2.277)	0.017	1.492 (0.994–2.240)	0.054
**NE**(×10^9^/L)	<7/≥7	1.764 (1.171–2.657)	0.007	1.080 (0.620–1.883)	0.785
**LY**(×10^9^/L)	<4/≥4	0.788 (0.250–2.482)	0.684	–	NI
**HGB**(g/L)	<100/≥100	0.869 (0.600–1.258)	0.458	–	NI
**PLT**(×10^9^/L)	<300/≥300	1.012 (0.699–1.466)	0.948	–	NI
**ALT**(U/L)	<40/≥40	1.424 (0.894–2.270)	0.137	–	NI
**AST**(U/L)	<45/≥45	1.279 (0.825–1.983)	0.272	–	NI
**ALP**(U/L)	<100/≥100	1.171 (0.680–2.017)	0.568	–	NI
**GGT**(U/L)	<50/≥50	0.953 (0.545–1.664)	0.867	–	NI
**ALB**(g/L)	<35/≥35	0.976 (0.629–1.514)	0.914	–	NI
**TBIL** (mmol/L)	<20.5/≥20.5	1.088 (0.701–1.688)	0.706	–	NI
**IBIL** (mmol/L)	<15/≥15	1.493 (1.030–2.166)	0.035	1.345 (0.895–2.021)	0.154
**CRP** (mg/L)	<8/≥8	1.189 (0.779–1.814)	0.422	–	NI
**Tumor** **differentiation**	high/ moderate / poor	2.029 (1.421–2.895)	<0.001	1.669 (1.126–2.476)	0.011
**Tumor size** (cm)	<2/≥2	1.082 (0.837–1.397)	0.548	–	NI
**Macrovascular Invision**	Absent/Present	1.998 (1.010–3.954)	0.047	1.521 (0.639–3.621)	0.344
**Microvascular Invision**	Absent/Present	1.592 (1.080–2.347)	0.019	1.098 (0.708–1.703)	0.678
**LN metastasis**	Absent/Present	1.545 (1.066–2.240)	0.022	0.640 (0.276–1.487)	0.299
**TNM Stage**	IA/IB/IIA/IIB/III	1.208 (1.051–1.388)	0.008	1.035 (0.777–1.379)	0.814
**CEA** (μg/L)	<5/≥5	1.356 (1.030–2.289)	0.035	1.172 (0.771–1.283)	0.457
**CA199**(U/mL)	<35/≥35	2.075 (1.289–3.339)	0.003	1.250 (0.745–2.096)	0.398
**Chemotherapy**	Absent/Present	0.816 (0.562–1.186)	0.286	–	NI
**LNMS**	N0/N1/N2	1.574 (1.199–2.067)	0.001	1.786 (0.882–3.618)	0.107
**PLNR**	≤0.167/>0.167	1.367 (0.870–2.150)	0.176	–	NI
**NLR**	≤3.32/>3.32	1.599 (1.104–2.317)	0.013	0.820 (0.399–1.683)	0.588
**dNLR**	≤1.94/>1.94	1.451 (1.002–2.101)	0.049	0.894 (0.478–1.673)	0.726
**PLR**	≤99.02/>99.02	1.389 (0.676–2.853)	0.371	–	NI
**PI**	0 / 1 / 2	1.216 (0.906–1.633)	0.193	–	NI
**PNI**	0 / 1	1.063 (0.693–1.632)	0.778	–	NI
**mGPS**	0/1/2	0.996 (0.784–1.264)	0.971	–	NI
**NLR-PI**	1 / 2 / 3	1.570 (1.192–2.068)	0.001	1.684 (1.015–2.796)	0.044

*NE* Neutrophilic granulocyte, *LY* Lymphocyte, *GGT* Glutamyltranspeptidase, *ALP* Alkaline phosphatase, *LNMS* Lymph node metastasis stage, *PLNR* Positive lymph node ratio. Other Abbreviations as in Table 1 and Table 2

### DFS and prognostic factors

**Fig. 2:**
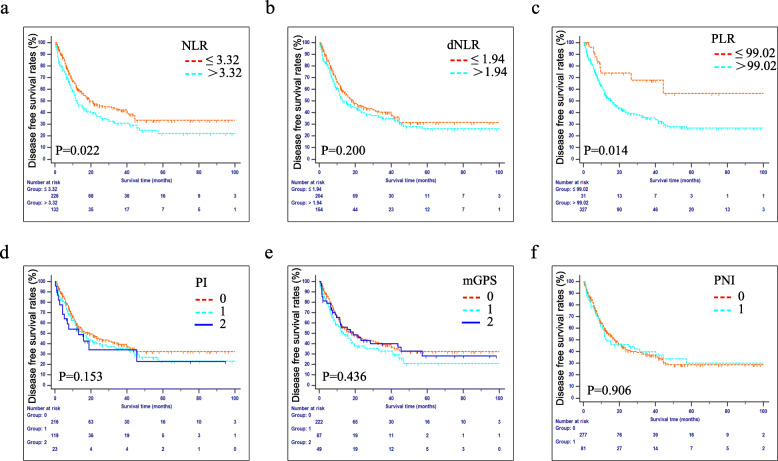
Kaplan-Meier curves of the DFS in patients who were diagnosed with AC after PD. Patients were stratifed according to each inflammation-based index. **a** NLR, **b** dNLR, **c** PLR, **d** PI, **e** mGPS and **f** PNI

The estimated 1-, 2-, and 3-year DFS rates for all patients were 58.0, 42.8, and 37.8%, respectively. The median DFS was 16.9 months. The correlations between the inflammation-based indexes and DFS are shown in Fig. 2. Elevated NLR (*P* < 0.05, Fig. 2a) and PLR (*P* < 0.05, Fig. 2c) were associated with reduced DFS. Nevertheless, dNLR, PI, mGPS and PNI failed to distinguish patients with longer DFS from those with shorter DFS (Fig. 2b, d-f).

Univariate survival analysis for DFS revealed significant associations between unfavourable DFS and higher pretreatment NLR (HR 1.406, 95% CI 1.051–1.879, *P* < 0.05) and PLR (HR 2.432, 95% CI 1.197–4.942, *P* < 0.05). Other significant prognostic parameters related to DFS included tumour differentiation, macrovascular invasion, lymph node metastasis, TNM stage, lymph node metastasis stage, CEA, CA19–9 and whether subsequent chemotherapy was administered. Multivariate Cox proportional hazards analysis showed that tumour differentiation (HR 1.593, 95% CI 1.185–2.141, *P* < 0.05) and whether subsequent chemotherapy was administered (HR 1.427, 95% CI 1.056–1.928, *P* < 0.05) were independent predictors of DFS (Table 4).

**Table 4:** Univariate and multivariate analyses of DFS

Characteristic	Parameter	Univariate analysis		Multivariate analysis	
		HR(95% CI)	***P***	HR(95% CI)	***P***
**Gender**	Female / Male	1.042 (0.776–1.399)	0.706	–	NI
**Age** (years)	<60/ ≥60	1.160 (0.866–1.555)	0.319	–	NI
**NE**(×10^9^/L)	<7/≥7	1.218 (0.864–1.717)	0.260	–	NI
**LY**(×10^9^/L)	<4/≥4	0.804 (0.330–1.955)	0.630	–	NI
**HGB**(g/L)	<100/≥100	1.019 (0.765–1.358)	0.897	–	NI
**PLT**(×10^9^/L)	<300/≥300	1.017 (0.763–1.356)	0.907	–	NI
**ALT**(U/L)	<40/≥40	1.395 (0.973–1.999)	0.070	–	NI
**AST**(U/L)	<45/≥45	1.224 (0.875–1.712)	0.238	–	NI
**ALP**(U/L)	<100/≥100	1.225 (0.798–1.881)	0.353	**–**	NI
**GGT**(U/L)	<50/≥50	0.926 (0.598–1.433)	0.729	**–**	NI
**ALB**(g/L)	<35/≥35	0.940 (0.665–1.330)	0.728	–	NI
**TBIL** (mmol/L)	<20.5/≥20.5	1.118 (0.790–1.582)	0.529	–	NI
**IBIL** (mmol/L)	<15/≥15	1.365 (1.024–1.821)	0.034	–	NI
**CRP** (mg/L)	<8/≥8	1.291 (0.925–1.801)	0.133	–	NI
**Tumor** **differentiation**	high/ moderate / poor	1.704 (1.296–2.240)	<0.001	1.593 (1.185–2.141)	0.002
**Tumor size** (cm)	<2/≥2	1.116 **(**0.914–1.363**)**	0.281	**–**	NI
**Macrovascular Invision**	Absent/Present	2.216 (1.260–3.896)	0.006	1.758 (0.834–3.704)	0.138
**Microvascular Invision**	Absent/Present	1.806 (0.897–1.245)	0.060	**–**	NI
**LN metastasis**	Absent/Present	1.874 (1.404–2.500)	<0.001	0.678 (0.363–1.266)	0.223
**TNM Stage**	IA/IB/IIA/IIB/III	1.074 (1.181–1.467)	<0.001	1.074 (0.848–1.361)	0.553
**CEA** (μg/L)	<5/≥5	1.291 (1.179–2.172)	0.003	1.291 (0.935–1.782)	0.121
**CA199**(U/mL)	<35/≥35	1.416 (1.351–2.848)	<0.001	1.416 (0.959–2.093)	0.080
**Chemotherapy**	Absent/Present	1.527 (1.145–2.036)	0.004	1.427 (1.056–1.928)	0.021
**LNMS**	N0/N1/N2	1.639 (1.432–2.161)	<0.001	1.639 (0.966–2.782)	0.067
**PLNR**	≤0.167/>0.167	1.178 (0.807–1.717)	0.396	–	NI
**NLR**	≤3.32 />3.32	1.406 (1.051–1.879)	0.022	1.450 (0.970–1.780)	0.078
**dNLR**	≤1.94/>1.94	1.207 (0.905–1.609)	0.200	–	NI
**PLR**	≤99.02/>99.02	2.432 (1.197–4.942)	0.014	2.040 (0.994–4.185)	0.052
**PI**	0 / 1 / 2	1.179 (0.941–1.479)	0.153	–	NI
**PNI**	0 / 1	1.021 (0.726–1.436)	0.906	–	NI
**mGPS**	0/1/2	1.077 (0.894–1.298)	0.436	–	NI
**NLR-PI**	1 / 2 / 3	1.304 (1.047–1.624)	0.018	1.285 (1.014–1.630)	0.038

Abbreviations as in Table 3

### Prognostic value of inflammatory indexes

**Fig. 3:**
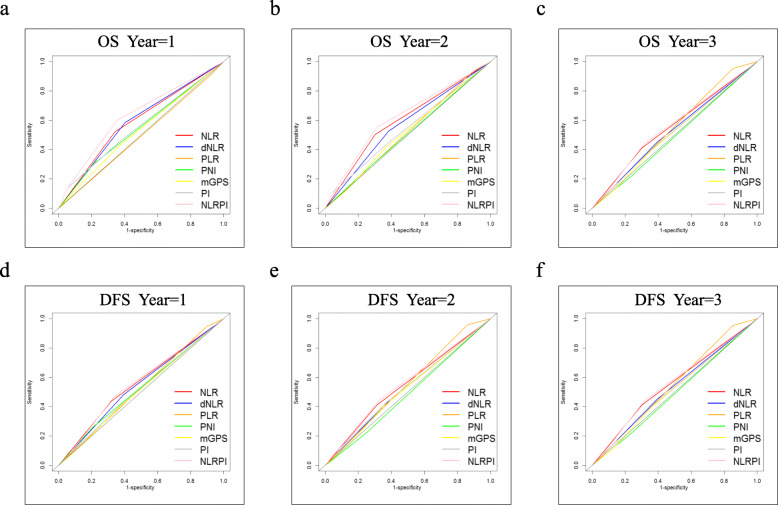
AUROC for OS and DFS stratifed by each inflammation-based index at 1-year, 2-year and 3-year. **a** OS at 1-year, **b** OS at 2-year, **c** OS at 3-year, **d** DFS at 1-year, **e** DFS at 2-year and **f** DFS at 3-year

Moreover, the prognostic values of the inflammation-based indexes for both OS and DFS were compared by analysing the AUROC values. The ROC curves for OS and DFS prediction were calculated for the patients at 1, 2, and 3 years of follow-up. More specifically, for OS, the AUROC values of the NLR and dNLR scores were consistently higher than those of most of the other inflammatory indexes; in addition, the NLR and dNLR scores were higher in patients at 1 year, and the NLR and PLR scores were higher in patients at 2 and 3 years of follow-up for DFS (Fig. 3) (Table 5).

**Table 5:** The comparison of the AUROC values among each inflammation-based scores

Characteristic	OS			DFS		
	Time			Time		
	Year = 1	Year = 2	Year = 3	Year = 1	Year = 2	Year = 3
**NLR**	0.642	0.659	0.619	0.664	0.651	0.656
**dNLR**	0.64	0.626	0.60	0.646	0.632	0.632
**PLR**	0.542	0.578	0.586	0.63	0.653	0.654
**PNI**	0.596	0.56	0.556	0.629	0.59	0.595
**mGPS**	0.578	0.578	0.528	0.612	0.623	0.615
**PI**	0.607	0.593	0.546	0.621	0.638	0.628
**NLR-PI**	0.712	0.704	0.704	0.724	0.719	0.72

Abbreviations as in Table 1

However, NLR or other factors alone only showed a moderate ability in predicting the prognosis of patients with AC after pancreaticoduodenectomy. From an overall perspective, in addition to NLR, PI had a higher AUROC values regardless of OS or DFS. To further enhance the diagnostic efficiency, a new inflammation-based scoring system was generated by combining NLR with PI. The NLR-PI score was defined as follows: (1) NLR-PI = 1: NLR = 0 and PI = 0; (2) NLR-PI = 2: NLR = 0 and PI = 1 or 2 or NLR = 1 and PI = 0; and (3) NLR-PI = 3: NLR = 1 and PI = 1or 2. Among the 358 patients, 217 (60.6%) patients had a low NLR-PI score (NLR-PI = 1) and 141 (39.4%) patients had an NLR-PI score of 2 or 3. In terms of the survival differences in patients with NLR-PI scores of 2 and 3, the median survival times for patients with NLR-PI scores of 2 and 3 were 16.5 and 32 months, respectively, and the survival differences were significant (*P* < 0.05). As shown in Table 5, the AUROC value of NLR-PI is the maximal among these indexes mentioned above under any conditions, which means that the NLR-PI score showed a better distinguishing power for predicting the prognosis of patients with AC who were treated with the Whipple procedure than the other inflammation-based indexes alone. In other words, with regard to both OS and DFS, the NLR-PI score divided patients into subgroups more precisely. Additionally, the concordance index (C-index) of each inflammatory parameter was calculated (Table 6) and compared with each other (Table 7). These results also verified that the NLR-PI score had a superior discriminative capacity.

**Table 6:** The C-index value of each inflammation-based score

Characteristic		NLR	dNLR	PLR	PI	PNI	mGPS	NLR-PI
**c-index** **Value** **(95% CI)**	**OS**	0.674 (0.624–0.724)	0.663 (0.613–0.713)	0.614 (0.589–0.639)	0.632 (0.577–0.687)	0.619 (0.572–0.666)	0.59 (0.539–0.641)	0.7 (0.647–0.753)
	**DFS**	0.651 (0.613–0.689)	0.603 (0.573–0.634)	0.627 (0.608–0.646)	0.634 (0.594–0.674)	0.609 (0.576–0.642)	0.627 (0.587–0.667)	0.657 (0.617–0.697)

Abbreviations as in Table 1

**Table 7:** The pairwise comparison of C-indexes of each inflammation-based C-index valuescores for OS and DFS prediction

Characteristic			NLR	dNLR	PLR	PI	mGPS	PNI	NLR-PI
***P*** **value**	**OS**	**NLR**	–	0.3357	0.0138	0.0857	0.0013	0.0197	0.1976
		**dNLR**	0.3357	–	0.0368	0.1643	0.0053	0.0611	0.0902
		**PLR**	0.0138	0.0368	–	0.2635	0.1817	0.4145	0.0011
		**PI**	0.0857	0.1643	0.2635	–	0.0659	0.3406	0.0104
		**mGPS**	0.0013	0.0053	0.1817	0.0659	–	0.1511	0.0001
		**PNI**	0.0197	0.0611	0.4145	0.3406	0.1511	–	0.0018
		**NLR-PI**	0.1976	0.0902	0.0011	0.0104	0.0001	0.0018	–
	**DFS**	**NLR**	–	0.3357	0.0101	0.0857	0.0150	0.0197	0.1976
		**dNLR**	0.3357	–	0.0303	0.1643	0.0335	0.0611	0.0902
		**PLR**	0.0101	0.0303	–	0.2573	0.4440	0.4145	0.0006
		**PI**	0.0857	0.1643	0.2573	–	0.2195	0.3406	0.0092
		**mGPS**	0.0150	0.0335	0.4440	0.2195	–	0.3799	0.0009
		**PNI**	0.0197	0.0611	0.4145	0.3406	0.3799	–	0.0018
		**NLR-PI**	0.1976	0.0902	0.0006	0.0092	0.0009	0.0018	–

Abbreviations as in Table 1

**Fig. 4:**
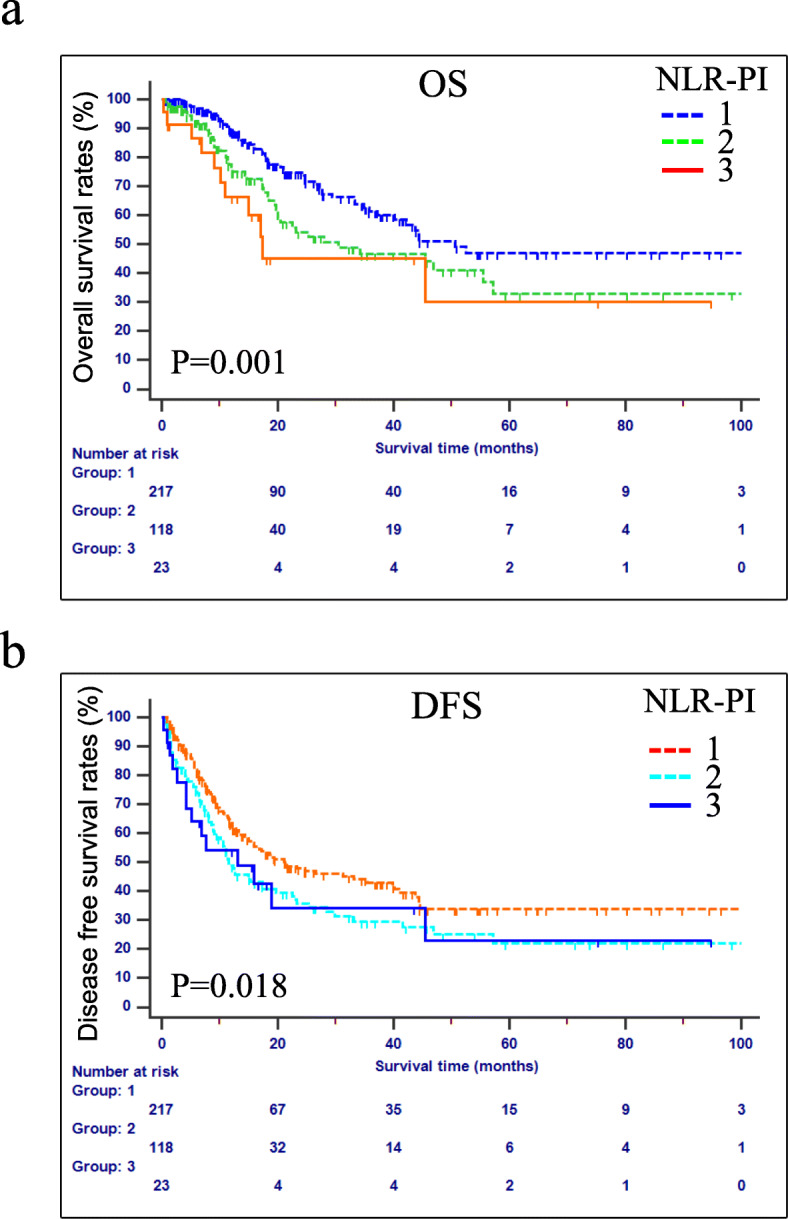
Kaplan-Meier curves of the OS and DFS according to NLR-PI score. **a** OS, **b** DFS

Furthermore, patients with low NLR-PI scores also had better long-term survival and DFS than patients with high NLR-PI scores (*P* < 0.05, Fig. 4). In univariate survival analysis, the NLR-PI score was also significantly associated with OS and DFS (OS: HR 1.570, 95% CI 1.192–2.068, *P* < 0.05; DFS:HR 1.304, 95% CI 1.047–1.624, *P* < 0.05). In multivariate Cox proportional hazards analysis, the NLR-PI score was also viewed as an independent predictor of both OS and DFS (OS: HR 1.684, 95% CI 1.015–2.2.796, *P* < 0.05; DFS: HR 1.285, 95% CI 1.014–1.630, *P* < 0.05) (Tables 3 and 4).

## Discussion

Although the immune and inflammatory responses of the host are strongly associated with the cancer progression, few researchers have paid sufficient attention to inflammation-based indexes. Until now, most researchers have focused on conventional factors, including age, grade, tumour size, lymph node ratio, extension range and so on, to establish a nomogram or only focus on a certain inflammatory factor for predicting prognosis [17]. In the present study, we took a more global approach to this problem. We systematically demonstrated the prognostic significance of preoperative inflammatory parameters, including NLR, dNLR, PLR, mGPS, PI and PNI, for predicting prognosis in a cohort of 358 patients who suffered from AC and had underwent the Whipple procedure as curative resection. To the best of our knowledge, this is a more comprehensive report in this field not only because we included a higher number of cases than former studies but also because we adopted multiple assessment methods, such as the AUROC value and the C-index.

According to our results, the NLR score was found to be an effective prognostic factor for both OS and DFS after surgical treatment. In addition, our study also showed that there was a correlation between dNLR and OS while PLR can prognosticate DFS in the overall patient cohort. More innovatively, we investigated a new inflammation-based score combining NLR and PI, namely, NLR-PI, which was found to be a powerful prognostic factor with superior discriminative capacity for predicting OS and DFS compared with other indicators. The cohort was divided into three groups according to the NLR-PI score, and patients in the higher NLR-PI score group were associated with poor prognosis. However, because the number of patients with an NLR-PI score of 3 was relatively small compared with that of patients with an NLR-PI score of 2, their survival plots seemed to be slightly closed, while the true difference in the survival time of these two groups was significant.

It is well known that the inflammatory state of patients quantified and characterized by various inflammatory factors is strongly associated with specific tumorigenesis and development [18]. Cancer-related inflammatory responses have extensive effects on the malignant biological properties of tumour tissue, including cellular proliferation, survival, angiogenesis, migration, invasion and metastasis [19, 20]. More precisely, in addition to inflammatory factors, which are expected to be promising indicators for the early diagnosis of neoplasia and play a key role in improving the prognosis of patients, malignant cells have tight cross-talk with the tumour immune microenvironment regulated by relevant cytokines and signal transduction [21]. For example, the inflammatory factor lipopolysaccharide (LPS), which activates Toll-like receptors (TLRs), increased the invasive behaviour and antiapoptotic effects of cancer by activating the transcription factor nuclear factor κB (NF-κB) signalling pathway. Although the occurrence mechanisms of unique systemic inflammation in cancer patients remain disputed, necrosis and local tissue damage together with the production of inflammatory mediators released by the cancer itself or leukocytes may be one of the prime reasons [22].

On the basis that systemic inflammation is responsible for cancer generation, invasion, metastasis and even resistance to chemotherapy or radiotherapy, growing evidence has shown that the measurement of some inflammatory markers has prognostic significance in cancer patients. A severe systemic inflammatory response is usually associated with poor prognosis in multiple types of carcinomas. It is worth noting that inflammation-based assessment tools based on inflammatory indexes have been developed and put into use in some institutions. With the emergence of immunotherapy, the immune system status of patients and the role of innate immunity-mediated inflammation in cancer biology have drawn great attention [23].

As a marker of systemic inflammation, several retrospective studies have confirmed that NLR is a reliable predictor of postoperative prognosis in patients with multiple types of tumours. Various systemic inflammation-based scoring systems, in which a certain preoperative NLR score acts as an independent prognostic factor, are supposed to be used for speculating the OS or DFS of patients after curative resection. For instance, Han et al. showed that pretreatment NLR was a prognostic index for patients with glioblastoma (GBM), and Weng et al. showed that the NLR level was associated with the different grades of gliomas [18]. Wang et al. suggested that albumin-NLR was a superior independent prognostic factor of OS for colorectal cancer patients who received radical resection in the multivariate survival analysis [24]. Moreover, these results were also verified in patients suffering from hepatocellular carcinoma. Elegant theories that reveal the reason for why elevated NLR is associated with poor prognosis remain unclear. However, several underlying mechanisms have been recognized. The elevation in NLR was significantly related to high neutrophil infiltration and low cytolytic activities of lymphocytes. Elevated neutrophils will produce more proangiogenic factors, including vascular endothelial growth factors (VEGFs) and matrix metalloproteinases, to stimulate tumour development and progression by enhancing vascularization. On the other hand, adaptive immune cells, such as B lymphocytes, T lymphocytes, CD8^+^ cytotoxic T lymphocytes and CD4^+^ helper T lymphocytes, have pivotal effects on the suppression of oncogenesis [25]. Therefore, the decreased lymphocyte count, which represents an insufficient immunologic reaction to the malignant tumour, consequently enables tumour progression and metastasis [22, 26]. Consistent with other research results and the immune dysregulation state represented by a high NLR score, in this study, we also discovered that a higher NLR score predicted a shorter OS and DFS which means a worse clinical outcome in patients with AC after pancreaticoduodenectomy.

Postoperative PLR has also been extensively researched as an evaluation tool for inflammatory and immune responses and is reportedly a novel prognostic factor in various malignancies. At present, the findings of many studies focusing on the prognostic role of PLR are contradictory. Lim et al. showed that a higher PLR was an independent predictor of shorter survival in stage IV non-small cell lung cancer (NSCLC) patients with cytologically proven malignant pleural effusion (MPE) [27]. However, different results were detected in the studies by Peng et al. and Kabir et al., which revealed that PLR was not evidently associated with the OS or recurrence-free survival (RFS) of patients with hepatocellular carcinomas (HCC) [28, 29]. In our study, although no significant difference between PLR and OS was found in patients who underwent the Whipple procedure as curative resection for periampullary carcinoma, PLR was a strong independent prognostic factor for DFS. Specifically, elevated PLR was associated with a shorter DFS. Theoretically, platelets can also release various growth factors, including VEGF and platelet-derived growth factor (PDGF), into the tumour microenvironment and promote tumour growth, migration and immune evasion [8, 30]. PI was proposed by G. Kasymjanova et al. and has been proven to be a reliable index for evaluating the prognosis of multiple kinds of tumours by others [23]. It is defined by the inflammatory markers C-reactive protein (CRP) and white blood cells (WBCs). In our study, we attempted to combine NLR with PI to enhance the prediction ability. This is the first study to evaluate the predictive efficacy of NLR-PI and compare it with other indexes for patients with AC after pancreaticoduodenectomy. Surprisingly, when we established NLR-PI as mentioned above, our results demonstrated that the combined score consistently exhibited higher AUROC values at 1, 2 and 3 years for OS and DFS compared to NLR or PI alone, and the Kaplan-Meier survival curves showed that the combined score divides patients into subgroups more accurately. NLR-PI is based on the level of neutrophil granulocytes, lymphocytes and CRP, which can be easily obtained. Among these indexes, CRP is a classical acute-phase protein displaying a rapid and pronounced rise in its plasma concentration in response to acute inflammation, infection, and tissue damage. It was reported that there is a positive association between elevated circulating CRP levels and the risk of cancer [31, 32]. The combination of NLR with PI was more comprehensive, and such a combination of inflammatory factors may contribute to a robust prognostic model for patients diagnosed with AC who underwent PD as curative resection.

This study has several limitations. First, this report had a retrospective study design, which may induce some selection bias and relied on a single institutional dataset. Second, some patients were administered routine adjuvant chemotherapy, but since the chemotherapy data were incomplete, a thorough analysis of the relationship between treatment agents and inflammatory factors could not be performed. Third, there are two subtypes of ACs, namely, pancreaticobiliary and intestinal subtypes. Some patients, especially those who underwent surgery in earlier years, cannot be specifically classified according to pathology, so all patients were included in our study as a whole cohort and analysed. Finally, there might be other reasonable cutoff values for variables from other studies and NLR-PI is a novel inflammation score; therefore, a large-scale prospective validation study is needed to confirm these results and validate this factor’s prognostic value and further applications.

## Conclusions

In conclusion, the predictive powers of several preoperative inflammation-based prognostic scores were assessed and compared in patients with AC who underwent PD as curative resection. Among these indexes, NLR was found to predict both the OS and DFS of patients, while dNLR or PLR was only related to one of them. More significantly, we proposed a novel factor, NLR-PI, which was a more effective and independent predictive factor for poor outcomes in patients. NLR, dNLR, PLR and NLR-PI have an inverse correlation with OS or DFS. Further prospective studies should be conducted to confirm these results and to provide evidence for individualized treatment.

## Data Availability

The datasets generated and/or analysed during the current study are not publicly available but are available from the corresponding author on reasonable request. The key raw data have been uploaded onto the Research Data Deposit public platform (www.researchdata.org.cn), with the approval RDD number of RDDA2020001529.

## Abbreviations

AC: Ampullary cancer
PD: Pancreaticoduodenectomy
ROC: Receiver operating characteristic
AUROC: Area under the time-dependent receiver operating characteristic curves
WBC: White blood cell count
HGB: Haemoglobin
PLT: Platelets
NE: Neutrophilic granulocyte
LY: Lymphocyte
ALT: Alanine aminotransferase
AST: Aspartate aminotransferase
GGT: Glutamyltranspeptidase
ALP: Alkaline phosphatase
ALB: Albumin
TBIL: Total serum bilirubin
IBIL: Indirect serum bilirubin
CRP: C-reactive protein
CEA: Carcinoembryonic antigen
CA19–9: Carbohydrate antigen 19–9
NLR: Neutrophil-to-lymphocyte ratio
dNLR: Derived neutrophil-to-lymphocyte ratio
PLR: Platelet-to-lymphocyte ratio
mGPS: Modified Glasgow Prognostic Score
PNI: Prognostic nutritional index
PI: Prognostic index
TNM: Tumour-node-metastasis
LN: Lymph node
LNMS: Lymph node metastasis stage
PLNR: Positive lymph node ratio
OS: Overall survival
DFS: Disease-free survival
RFS: Recurrence-free survival
HCC: Hepatocellular carcinomas
SII: Systemic immune inflammation index
CT: Computed tomography
MR: Magnetic resonance
EUS-FNA: Endoscopic ultrasonography-guided fine needle aspiration
HR: Hazard ratio
LPS: Lipopolysaccharide
TLRs: Toll-like receptors
GBM: Glioblastoma
VEGFs: Vascular endothelial growth factors
PDGF: Platelet-derived growth factor
MPE: Malignant pleural effusion
NSCLC: Non-small cell lung cancer
